# The Making of a New Medical Specialty: A Policy Analysis of the Development of Emergency Medicine in India

**DOI:** 10.15171/ijhpm.2018.55

**Published:** 2018-07-11

**Authors:** Veena Sriram, Adnan A. Hyder, Sara Bennett

**Affiliations:** ^1^Center for Health and the Social Sciences, University of Chicago, Chicago, IL, USA.; ^2^Health Systems Program, Department of International Health and International Injury Research Unit, Johns Hopkins Bloomberg School of Public Health, Baltimore, MD, USA.; ^3^Health Systems Program, Department of International Health, Johns Hopkins Bloomberg School of Public Health, Baltimore, MD, USA

**Keywords:** India, Emergency Medicine, Agenda-Setting, Medical Specialization, Health Policy

## Abstract

**Background:** Medical specialization is an understudied, yet growing aspect of health systems in low- and middleincome countries (LMICs). In India, medical specialization is incrementally, yet significantly, modifying service delivery, workforce distribution, and financing. However, scarce evidence exists in India and other LMICs regarding how medical specialties evolve and are regulated, and how these processes might impact the health system. The trajectory of emergency medicine appears to encapsulate broader trends in medical specialization in India – international exchange and engagement, the formation of professional associations, and a lengthy regulatory process with the Medical Council of India. Using an analysis of political priority setting, our objective was to explore the emergence and recognition of emergency medicine as a medical specialty in India, from the early 1990s to 2015.

**Methods:** We used a qualitative case study methodology, drawing on the Shiffman and Smith framework. We conducted 87 in-depth interviews, reviewing 122 documents, and observing six meetings and conferences. We used a modified version of the ‘Framework’ approach in our analysis.

**Results:** Momentum around emergency medicine as a viable solution to weak systems of emergency care in India gained traction in the 1990s. Public and private sector stakeholders, often working through transnational professional medical associations, actively pursued recognition from Medical Council of India. Despite fragmentation within the network, stakeholders shared similar beliefs regarding the need for specialty recognition, and were ultimately achieved this objective. However, fragmentation in the network made coalescing around a broader policy agenda for emergency medicine challenging, eventually contributing to an uncertain long-term pathway. Finally, due to the complexities of the regulatory system, stakeholders promoted multiple forms of training programs, expanding the workforce of emergency physicians, but with limited coordination and standardization.

**Conclusion:** The ideational centrality of postgraduate medical education, a challenging national governance system, and fragmentation within the transnational stakeholder network characterized the development of emergency medicine in India. As medical specialization continues to shape and influence health systems globally, research on the evolution of new medical specialties in LMICs can enhance our understanding of the connections between specialization, health systems, and equity.

## Background

Medical specialization is an understudied, yet growing aspect of health systems in low- and middle-income countries (LMICs).^1^ In LMICs, a tension has emerged between the growing presence of medical specialists, and the need for more equitable health systems.^1^ On the one hand, specialization has emerged as a dominant value in the medical profession, incentivizing students and doctors to specialize, and modifying patterns of service delivery.^2^ On the other hand, specialization has resulted in a disproportionate focus on hospital-based specialized care, often at the expense of primary care approaches that can benefit the poor and disenfranchised.^1,3^

Social science explorations highlight a host of technical, political, social, and economic factors driving the emergence of new medical specialties in high-income settings.^4-6^ Specialization is a feature of many professions, with professional bodies being comprised of ‘segments’ based upon mission, methodologies, clients, and interests.^7^ Focusing on the formation of medical specialization, Leeming^6^ and Döhler^5^ categorize influencing variables as follows: (1) conceptual and technological innovation; (2) intra-professional competition driven in part by market forces; (3) social and political factors; (4) structural and organizational aspects of academic medicine and health service delivery. The evolution of medical specialties in high-income settings, particularly in the nineteenth and early twentieth century, was lengthy, complex and at times contentious.^4,8,9^ Scholars have highlighted the roles of multiple stakeholders (notably national professional associations) and the importance of international exchange, in advancing and standardizing specialization.^4,6,8-10^

Medical specialization also raises key policy questions for the health sector, including how and why new medical specialties are formally recognized.^4^ For example, in high-income settings, organized medicine appears to have played a pivotal role in formalizing medical specialties, initially by leading the effort to create regulatory systems that enabled such recognition and later, by promoting new specialties within established regulatory systems.^4,8,9,11^ More broadly, the state has had a more limited role in specialization policy in high-income settings. While the state has occasionally, where permissible, promoted the recognition of particular specialties, it has more frequently played an indirect role, for example, through its funding and administration of health delivery systems.^4^

Medical specialization in LMICs, settings with different forms of organized medicine, regulation, and state intervention, remains under-studied and under-theorized.^12,13^ In India, medical specialization is increasingly impacting the structure of health service delivery and medical education, arguably driven by underlying shifts towards tertiary care and privatization.^14-17^ Recent broad specialties include infectious diseases, palliative medicine and emergency medicine.^18,19^ From a policy standpoint, the available literature suggests a complicated regulatory architecture for medical specialization in India, and challenges in acquiring recognition for new medical specialties from the Medical Council of India, a statutory professional council comprised of doctors that by Act of Parliament oversees medical colleges.^20-22^ There also appears to be a prominent role for specialist doctors from high-income countries in promoting new specialties in the country.^23,24^ Insider accounts of the process to promote new medical specialties also provide valuable insight into the political nature of the policy process for specialization,^20,21^ and other experts have signaled the need for in-depth, systematic research on specialization in India.^25^

We have conducted an analysis of political priority setting for medical specialization to unpack the emergence and recognition of new medical specialties. The trajectory of emergency medicine appears to encapsulate broader trends in medical specialization in India – international exchange and engagement, the formation of professional associations, and a lengthy regulatory process.^20,23,26,27^ Using a qualitative case study methodology, our objective is to examine the emergence and recognition of new medical specialties in India, by exploring how and why the issue of emergency medicine as an academic specialty gained political priority with Indian regulators, specifically Medical Council of India.

### Conceptual Framework

To design and analyze this case, we looked towards theoretical frameworks regarding prioritization of policy issues. We utilized a framework on issue ascendance in global health from Shiffman and Smith^28^ that has been applied to several health policy analyses in global health.^29-32^ We decided to empirically test the applicability of this framework, as we believed that the main determinants were present in the evolution of emergency medicine in India, based on our *a priori* knowledge of the case.^28,33^ The framework and its subsequent iterations have drawn largely upon theory related to collective action and social constructionism.^34,35^ The original framework proposes four determinants for priority setting – actor power, ideas, political context and issue characteristics. Actor power draws from collective action theory, where concepts such as network cohesion, individual leadership, institutions and organizations with clear mandates to spearhead advocacy around a policy agenda have been previously identified as key factors for successfully gaining priority for policy issues.^34^ The nature of the policy network advancing prioritization also impacts the policy process, and several types have been put forward – for example, policy communities comprising smaller membership with more frequent, higher-quality interaction, issue networks of loosely connected members with fluctuating levels of interaction, and epistemic communities of professionals connected by similar expertise and shared educational and professional backgrounds.^36,37^ The determinant of ideas emerges from both collective action theory and social constructionism, in that groups negotiate and coalesce around internal or external ‘frames,’ or understanding of problems and solutions, but also that these ideas are fundamentally shaped by the lens of knowledge, culture and norms through which they are viewed.^34,35^ Issue characteristics also draws from social constructionism in that the features of the problem and proposed policies are similarly viewed through the social norms of the actors advancing the issue.^34^ Political context envelops the entire policy process, with windows of opportunity potentially emerging for priority setting to gain traction, and national and global institutions setting the ‘rules of the game’ for the policy-making process.^34^

We utilized a version of the framework that incorporated modifications from previous analyses, including a broader category on the power of ideas that builds on social constructionism by merging ideas and issue characteristics,^34,35^ the addition of a category for policy outcomes, and the adaptation of some factors from the global-level to the national-level (Table 1).^33^ Our goal was to understand each of these categories in a ‘garbage can’ approach by examining the ‘intermeshing’ of actors, ideas and context, rather than determining a particular order in which these factors contributed to political priority.^38^

**Table 1:** Conceptual Framework Guiding Study Design and Analysis^28,33,34^

Category	Description	Factors Shaping Political Priority
Actor power	The strength of the individuals and organizations concerned with the issue	Factors under this category include: 1. Policy community cohesion: The degree of coalescence among the network of individuals and organizations centrally involved with the issue 2. Leadership: The presence of individuals capable of uniting the policy community and acknowledged as particularly strong champions for the cause 3. Guiding organizations: Effectiveness of organizations with a mandate to lead the initiative 4. Civil society mobilization: The extent to which grassroots organizations have mobilized to press political authorities to address the issue 5. Policy networks involved in this case potentially include policy communities, epistemic communities, and issue networks
Power of ideas	The ways in which actors understand and portray the issue	1. Internal framing: The degree to which the policy community agrees on the definition of, causes of and solutions to the problem 2. External framing: Public portrayals of the issue in ways that resonate with external audiences, especially the political leaders who control resources 3. Features of the problem: Severity of the burden, and availability of suitable interventions
Political contexts	The environments in which actors operate	1. Policy windows: Political moments when conditions align favorably for an issue, presenting opportunities for advocates to influence decision makers 2. National governance structure: The degree to which norms and institutions operating in a sector provide a platform for effective collective action
Outcome	The level to which the issue has reached the policy agenda	The making of authoritative decisions and allotment of resources to the issue by policy-makers

## Methods

We utilized case study methodology to conduct this study due to its utility in understanding contemporary, complex social phenomena in real-life contexts.^39^ Based on our *a priori* understanding of emergency care in India, and following a literature search of the evolution of new medical specialties in India, we selected emergency medicine in India as a representative case of the emergence of ‘new’ medical specialties in India. In this paper, we will focus on the period of its emergence in the early 1990s leading up to its recognition in 2009, and briefly touch upon issues faced by the specialty from 2009 until 2015.

### Data Collection

Three forms of data collection were used iteratively for this study – in-depth interviews, document review, and non-participant observation.

*In-depth interviews:* We selected potential respondents through two forms of purposive sampling – maximum variation, whereby we attempted to capture similarities and differences in perspectives across a diverse group of stakeholders, and snowball sampling, where we requested respondents to share suggestions for information-rich respondents.^40^ We conducted data collection from March 2015 to March 2016, with the majority of interviews taking place in-person in India in 11 primarily metropolitan locations. We conducted a total of 87 interviews with 76 respondents (Table 2), completing the interviews when we had interviewed a majority of the key stakeholders in the case, and also captured an adequate diversity of viewpoints. Six individuals declined to be interviewed (several of whom were current or former regulatory and government stakeholders), and two individuals did not respond to requests for interviews. 72 interviews were conducted in-person, seven over the phone, and eight by Skype. Verbal consent was obtained from all respondents and a majority of interviews (n = 64) were audio-recorded. Interviews were semi-structured, and interview guides were designed with reference to the guiding conceptual framework. Examples of interview questions are provided in Table 3. Interviews were typically one hour in length. During each interview, we took extensive handwritten notes and summarized the content of the notes in the form of memos. Audio-recorded interviews were transcribed verbatim by a contracted transcriber. Each respondent was assigned a code during analysis. In order to protect the respondents’ identities, we have reported the broad categorizations to which the repondents belong (Table 3) and have withheld their organizational affiliation, location, and demographic characteristics.

**Table 2:** Number and Categorization of In-Depth Interview Participants

**Organizational Categorization **	**No. of Respondents**
Current and former central government officials	3
Current and former regulatory institutions officials	12
Development partners officials	2
Indian emergency medicine professionals	33
International emergency medicine professionals	14
Medical college leadership	6
Other new medical specialties stakeholders	5
Media representatives	1
Total	76

**Table 3:** Examples of Interview Questions Used in the Study

**Respondent Category**	**Examples of Interview Questions**
Indian stakeholders	1) How did you get involved with developing emergency medicine in India? 2) How did you get involved in efforts to formally gain approval for a specialty with Medical Council of India? 4) Thinking more broadly about emergency medicine specialization, why do you think that this idea gained importance in India? 5) How did stakeholders come to recognize that there was a problem with emergency care in India? 6) How do you think international actors have played a role in the development of emergency medicine? 7) What were the steps involved with requesting approval of emergency medicine as a specialty from Medical Council of India? 8) Which actors were primarily involved in the policy process? 9) What was different about Medical Council of India in 2009 as compared to 2000 when individuals had advocated for inclusion? 10) Could you describe the historical context in which emergency medicine became a specialty?
High-income country stakeholders	1) Besides India, are there other countries where you work to promote emergency medicine? 2) How did you get involved with developing emergency medicine in India? 3) How do you think international actors have played a role in the specialization of emergency medicine? 4) Thinking more broadly about emergency medicine specialization, why do you think that this idea gained importance? 5) What have you learnt from this process of developing emergency medicine as a specialty in India? 6) What impact do you think this specialty will have in India?
Regulators	1) What are the decision-making criteria that Medical Council of India utilizes for recognizing a new specialty? 2) When did you first become aware of requests for the inclusion of emergency medicine as a specialty in India? Who do you remember as being the main advocates? 3) Could you explain some of the reasons for and against inclusion of emergency medicine that were discussed by the committees involved? 4) What were the deciding factors by which the relevant committees decided to approve emergency medicine in 2009? 5) How has the experience of the development of emergency medicine been similar or different compared to other new specialties?

*Document Review:*

We sourced 122 documents for this study from a desk review, and through asking respondents to refer us to critical documents. Document categories included meeting minutes of key stakeholders, policy documents, correspondence between organizations, conference reports and brochures, and articles from Indian newspapers and magazines. Documents were analyzed for their relevance to the development of emergency medicine from the 1980s until 2015. Relevant summarized information was entered into a case study database on Microsoft Excel that allowed us to track our evidence.^39^

*Non-participant observation:* We utilized maximum variation and snowball sampling to purposefully select settings for observation.^40^ We observed a total of six meetings – three national-level emergency medicine conferences, two expert meetings on emergency care, and one state-level conference on health systems. We obtained permission from organizers of these conferences to observe the proceedings, and took extensive handwritten notes, which were later summarized as memos.

### Analysis

For the analysis, we utilized a version of the ‘framework’ method, a common analytic approach in policy research.^41^ The coding approach combined inductive and deductive approaches.^41^ We first developed initial codes based on the conceptual framework, and then built on this list by reviewing memos generated from the interviews, observations and select documents. We then conducted paper-based line-by-line coding on six transcripts, from which certain codes were inductively generated.^42^ Using Atlas.ti (version 1.0.24), we applied the new codebook to an additional seven transcripts, and based on this process, developed a final codebook. Using Atlas.ti, we applied this codebook to an additional 33 transcripts that were selected for in-depth coding due to the richness of the data presented in those interviews. We analyzed data according to the four broad analytic categories (Table 1) and generated sets of themes. We then entered the existing themes into a matrix, and reviewed the remaining 41 interview transcripts and/or memos, relevant documents, and observation data, in order to confirm or disconfirm themes, and present new information wherever possible.^43^ Finally, we engaged in respondent validation with three key informants by discussing certain findings and incorporating their feedback into the analysis during in-person meetings in September 2016.^44^ We also shared drafts of the findings with four respondents between January and March 2017 and incorporated feedback wherever appropriate. We sought feedback from respondents representing different professional associations and geographic regions within India in order to capture diversity in viewpoints.

### Reflexivity

Our approach was underpinned by a constructivist epistemology, whereby multiple truths and viewpoints emerge during the research process.^45^ The first author is an Indian origin female, who was enrolled in a doctoral degree program in a US-based institution at the time of the study. The first author collected data for the study, and regularly maintained reflexive memos noting possible biases and experiences at each stage of the research process.

## Results

We begin this section by presenting a brief overview of the development of emergency medicine in India (Table 4). We then present our findings in the following framework categories – actor power, the power of ideas, and political context. We conclude this section with a brief description of the policy outcomes of this case.

**Table 4:** Chronology of Key Events in the Trajectory of Emergency Medicine (1991–2015)

**Year**	**Key Milestones**	**Contextual Factors**
1991		Government of India policies for economic liberalization initiated
1992	All India Institute of Medical Sciences initiates plans for postgraduate program in emergency medicine	
1994–2000	Establishment of emergency departments in private hospitals and medical colleges, including Christian Medical College (Vellore), St. Johns Medical College (Bengaluru), Sri Ramachandra Medical College (Chennai), Sundaram Medical Foundation (Chennai) and Apollo Hospitals (Hyderabad)	
1998	First fellowship in emergency medicine offered at Christian Medical College, Vellore	
1999	First national conference and formal inauguration of Society for Emergency Medicine, India	
2000	Academy of Traumatology (Gujarat) is established	
Jan 2001		Bhuj earthquake (Gujarat)
Feb 2001	American Academy of Emergency Medicine in India is established	
2005	First Indo-US Emergency and Trauma Collaborative Summit takes places in Delhi	
2005	First international residency-style program in emergency medicine organized by Apollo Hospitals, Hyderabad, in coordination with Royal College of Emergency Medicine (UK)	
March 2007	National Human Rights Commission sends recommendations on emergency medicine from National Review on Health	
July 2007	First Masters of Emergency Medicine program offered at Malabar Institute of Medical Sciences (Kerala)	
July 2009	Medical Council of India approves emergency medicine as 29^th^ specialty	
May 2010		Medical Council of India is dissolved by President’s Order
2010	BJ Medical College and NHL Medical College (Gujarat) granted permission to begin MD programs in emergency medicine	Board of Governors instituted
2013	National Board of Examinations recognizes emergency medicine	Medical Council of India reinstated
July 2014–Dec 2015	Medical Council of India delays recognition for 11 permitted MD programs	

### Emergence of Emergency Medicine in India

The development of emergency medicine as a medical specialty in India grew out of a perceived need to improve weak systems of emergency care in both public and private sectors.^18,20,46^ Many respondents highlighted major challenges with all hospital-based emergency care in India, including poor quality, limited coordination, and inadequate prioritization. Exacerbating these issues was the staffing of Emergency Departments; typically, doctors handling emergencies were considered the least qualified to handle such cases, even though those patients often required serious attention.

*“[The Emergency Department], that is the area which is full of chaos, maximum chaos. One, because of large number of people who are coming there, secondly because most of the patients are critically ill, thirdly because the systems may not always be very much in functional state and fourthly, because many of these cases are also medico-legal in nature, so there is a rush of the people who are say from the law enforcing agency, police personnel etc. And also because the people who are taking care of these patients, they are not so well qualified, they are not so well equipped”* [Public sector medical college stakeholder].

These issues caught the attention of medical professionals during the 1980s, and possibly earlier, resulting in actions such as the initiation of short-course training programs in trauma care.^47,48^ However, emergency medicine as a viable solution to challenges with emergency care in India appeared to gain momentum in the 1990s. In high-income countries, emergency medicine emerged as a solution to similar issues, starting with the United States in the 1960s.^11^ As the field gained traction in North America, Europe and Australia, a few examples of diffusion to India appeared in the early 1990s, in both public and private health sectors^48^ The All India Institute of Medical Sciences (AIIMS) in New Delhi, the apex public institution for research, teaching and innovation in medical education in the country, began considering the establishment of a formal emergency medicine training program in 1992, inspired by a staff member who had recently returned from an international exchange program.^49^ In the private sector, Apollo Hospitals, an Indian healthcare company founded in 1983, took an interest in the specialty, driven in large part by the exposure of its leadership to healthcare in the United States. Apollo began to make connections with other interested stakeholders in India from the early 1990s, and authorized the establishment of formal Emergency Department services in the Apollo system in the mid-1990s.^50^ Sporadic efforts to establish Emergency Departments in other private hospitals also began in the early 1990s.^51^ Facilitated by exposure of their staff to emergency medicine in high-income countries, private medical colleges in the southern states of Karnataka and Tamil Nadu began establishing Emergency Departments and short-course training programs from 1994 onwards.^20^ Finally, the Bhuj earthquake in the western state of Gujarat in 2001 spurred specialist doctors from both public and private sectors in the state to actively explore options for improving emergency care, resulting in the formation of the Academy of Traumatology. Together with Gujarati diaspora based in the United States, this group advanced system-wide emergency care reform in the state, including legislation, pre-hospital emergency care, short-course training programs in hospital-based care and later, postgraduate training in medical colleges.

Supplementing, and in some cases, contributing to, the motivations of these actors were broader social and economic factors that played a key role in the development of the specialty. A critical factor underlying the growth of emergency medicine was the unprecedented shift towards privatization in the Indian healthcare market during the 1980s and 1990s, facilitating growth of ‘corporate’ hospitals, including Apollo.^52^ These hospitals, along with an explosion in the numbers of private nursing homes and hospitals, led to hospitals differentiating themselves through specialist care, including emergency medicine.^53,54^ The growth in specialist care impacted the decision making of medical students, leading to more students selecting specialization and super-specialization.^55^ Furthermore, a series of natural and manmade disasters in the 2000s, such as the Bhuj earthquake and the Indian Ocean tsunami in 2004, conveyed a growing perception that the country’s emergency response systems were deficient. During the mid-2000s, pre-hospital care began to gain policy-maker attention in multiple states, driven by policy efforts to establish statewide ambulance services, and augmented by a broader push during the same time period from the central government, the Centers for Disease Control and Prevention (CDC) and others to enhance road safety and trauma care in India.^56^ Other factors facilitating the growth of emergency medicine include an increase in employment opportunities for Indian graduates to work in Emergency Departments in high-income countries.^57^

### Actor Power

Groups of specialist doctors and aspiring emergency physicians dominated the landscape of actors promoting emergency medicine in India (Table 5). In 1999, the first national professional association, the Society for Emergency Medicine, India (SEMI), was formed. SEMI was given early support by Apollo, and its members included representatives from private sector hospitals and medical colleges. The association allowed for likeminded individuals to pursue a policy and training agenda for the specialty, including formal recognition with regulators. From 1990 and possibly earlier, emergency physicians (including those of Indian origin) from high-income countries, such as the United Kingdom and the United States, had periodically promoted emergency care in India, most commonly through short-course training programs.^48,58^ In 2001, emergency physicians and other doctors of Indian origin in the United States formally established a group called the American Association for Emergency Medicine in India (AAEMI) to support the development of emergency medicine in India, largely through partnership with SEMI. AAEMI members, along with other emergency physicians from the Indian diaspora in the United States, the United Kingdom, Australia, and Singapore, became actively involved in India, developing technical content for national and sub-national scientific conferences, and initiating training programs with Indian private sector hospitals.^23,59-61^ Several respondents from the diaspora noted the perceived advantages of their involvement, including credibility and technical expertise. Members of the diaspora also brokered the engagement of other high-income country stakeholders, such as non-Indian origin emergency physicians and professional associations, including the International Federation of Emergency Medicine, the Royal College of Emergency Medicine, and the American College of Emergency Physicians.

**Table 5:** Key Stakeholders and Roles in the Development of Emergency Medicine in India (1990–2015)

**Stakeholders**		**Key Roles**
Indian/transnational professional associations and organizations	SEMI	• Promoted recognition of emergency medicine as a medical specialty with Medical Council of India and National Board of Examinations • Supported development of formal and informal postgraduate training programs in private hospitals • Organized scientific conferences and other programs to raise awareness regarding emergency medicine
	Indo-US Emergency and Trauma Collaborative^62^	• Promoted recognition of emergency medicine as a medical specialty with Medical Council of India • Supported development of training programs in medical colleges • Engaged with public sector stakeholders around emergency care policy and research • Organized scientific conferences and other programs to raise awareness regarding emergency medicine
	Academy of Traumatology	• Organized activities in the state of Gujarat and in other parts of India around pre-hospital emergency care and short-course training programs in hospital-based emergency care • Initiated proposals for emergency medicine postgraduate training in state medical colleges, and advocated with Medical Council of India for formal recognition
	Other organizations (ie, Association for Trauma Care of India)	• Organized activities around pre-hospital and hospital-based emergency care
High-income country organizations and institutions	AAEMI	• Supported knowledge sharing through scientific conferences • Provided a platform from which AAEMI members engaged in developing institutional partnerships between Indian hospitals and their home institutions • Conducted some advocacy with regulators regarding formal recognition
	International professional associations (ie, International Federation of Emergency Medicine, Royal College of Emergency Medicine, American College of Emergency Physicians)	Supported knowledge sharing through scientific conferences and other fora Provided technical guidance to certain professional associations Partnered with Indian hospitals around training programs, certification, etc.
	Other diasporic organizations (ie, American Association of Physicians of Indian Origin)	Supported development of emergency medicine in India through scientific conferences, training programs, etc. Conducted advocacy for regulators regarding formal recognition
	Medical institutions	Partnered with Indian medical institutions to develop short- and long-term EM training programs
Regulatory institutions	Medical Council of India	Provided formal regulatory approval for initiating emergency medicine training programs in medical colleges
	NBE	Provided formal regulatory approval for initiating emergency medicine training programs in private hospitals
Government agencies	State Departments of Health	Facilitated approvals for emergency medicine training programs in public medical colleges
	Ministry of Health and Family Welfare	Provided final approval for recognition and for the initiation of emergency medicine training programs in medical colleges
	National Human Rights Commission	Promoted recognition of emergency medicine as a medical specialty with Medical Council of India (independent of professional associations)
Medical institutions	Private and public sector medical colleges	Initiated short- and long-term emergency medicine training programs Advocated with Medical Council of India for recognition Participated in professional associations for emergency medicine
	Private sector hospitals	Initiated formal and informal emergency medicine training programs Supported the growth of professional associations, particularly SEMI

Abbreviations: SEMI, Society of Emergency Medicine, India; NBE, National Board of Examinations; AAEMI, American Association for Emergency Medicine in India.

However, as the 2000s progressed, conflict emerged within SEMI, and between some members of SEMI and AAEMI. Most respondents agreed that key points of conflict were the role of the private sector and the influence of international actors. Some private sector hospitals and emergency physicians from high-income countries, and a few medical colleges, began ‘unregulated’ postgraduate training programs, ie, residency-style programs that did not have formal regulatory sanction. Many of these programs existed in an ambiguous regulatory zone due to a perceived lack of clear guidelines in the regulatory framework for postgraduate training programs in the private sector that did not fall within the purview of the two main regulators. However, some stakeholders felt that academic training programs within the formal regulatory framework in India, such as medical colleges, needed to be promoted.

*“People come from outside, run their own systems. There are many universities in America and even the [X Institution in the UK] come to India and they run their own sweatshops in various specialties, they award their own degrees. I have never seen an Indian University coming to America or UK and giving degrees to its citizens. But because our country’s system is so open and broad these guys can venture out into Indian soil and start distributing the diploma so that is colonization of academics according to me and it is going on very actively in India right now”* [High-income country emergency physician].

The role of international stakeholders was also debated within SEMI, in particular, the role of American stakeholders.

*“So there are many who resented the so called Americanization of emergency medicine for India and the Americans coming in to organize a program in India. Of course the bulk of Americans coming in were those of Indian origin but there were some who were not of Indian origin, of US origin who were doing it and they were given the prominence in many of these meetings. So you see, resentment was developing and so politics ruled SEMI for quite a few years. It was sad because that has slowed down the development of emergency medicine in India…”* [High-income country emergency physician].

Some respondents also noted that personal conflicts and underlying institutional rivalries contributed to conflicts within the network.

These disagreements resulted in a permanent fracturing of the network, with the formation of a parallel group, the Indo-US Emergency and Trauma Collaborative,^62^ a partnership comprised of medical colleges and high-income country medical institutions. This group decided to focus largely on developing training programs in medical colleges and promoting emergency medicine with government agencies. The involvement of AIIMS in INDUS-EM was considered by several respondents to be highly advantageous for that group, given the enormous technical and bureaucratic power held by AIIMS within India. Each group also became linked to a sectoral identity – INDUS-EM being more focused on the public sector, while SEMI focused on the private sector – despite the fact that the membership of each group came from both public and private sectors.

*“…the idea of two [associations] are different. The mentality and what they want to [do]. So that’s why….one is on the government side and other is on the corporate side”* [Indian emergency physician].

The groups also engaged with different global actors; for example, SEMI became the sole representative of India to the International Federation of Emergency Medicine, while INDUS-EM engaged with global health organizations such as the CDC and the World Health Organization (WHO). The groups also appeared to split on regional lines, with SEMI working more actively in South India, while INDUS-EM was more present in Northern states. The Academy of Traumatology continued to work largely independently in the state of Gujarat and in other parts of India. More broadly, state- and local-level efforts to develop emergency medicine took place at medical institutions across the country, sometimes in partnership with institutions in high-income countries, and in certain cases with limited or no input from the major associations.

Part of the challenge in building cohesion was the lack of unifying leaders for emergency medicine in India. Respondents appeared split in their acknowledgement of individual leaders for the development of the field in India. Several stakeholders were identified as the ‘father’ or ‘mother’ of emergency medicine in India, with only a few respondents noting the contributions of individuals outside their own sub-network. One respondent described the lack of a unifying leader in the following terms.

*“… the Critical Care Society speaks with a very cohesive central voice. That’s the one difference between them and us. They speak with a single voice, they iron out all the differences and they are able to say that this is the voice of Critical Care. We don’t have that; we don’t have a strong voice for Emergency Medicine in India, neither a person nor an organization”* [Private sector hospital stakeholder].

The relationship between the two associations was often contentious. Some respondents noted that the divide in the network was detrimental to the growth of the field, while a few others took a more optimistic view, noting that multiple professional associations for a single specialty was common in India.

### Power of Ideas

Despite the lack of network cohesion, stakeholders shared many of the same norms and beliefs – an understanding that emergency care systems in India were weak, and that introducing emergency medicine to India was the optimal solution. Further, these stakeholders also shared the same primary policy objective – the recognition of emergency medicine by the primary regulator of medical education in the country, Medical Council of India. Respondents discussed the implicit and explicit choices that stakeholders made, resulting in convergence around the idea of Medical Council of India recognition.

1) *Generation of specialists:* Some respondents described a straightforward argument for pursuing Medical Council of India recognition – formally trained specialists are required to develop a specialty. Some respondents noted that the long-term growth of the emergency physician workforce depended on having the Medical Council of India ‘stamp’ to facilitate interest amongst young doctors, and to ensure long-term career opportunities. A few respondents contested the focus on postgraduate education, noting that integrating emergency medicine into the undergraduate curriculum should have been a higher priority.

2) *Forging an identity:* Some respondents also noted that an underlying factor for seeking recognition from Medical Council of India was the need to forge an identity, and to gain respect in the medical profession. Broadly, respondents expressed the notion that formal recognition of the specialty was a key milestone in achieving a distinct identity for emergency medicine that encourages cohesiveness, and facilitated the entry of younger doctors.

*“…if you belong to a religion, and you don’t have a church, you don’t have a temple and you don’t have a flag then, what religion are you? How do you create your identity?”* [Former public sector hospital stakeholder].

3) *Norms of medical education in India:* Underlying the advocacy was the apparent need expressed by some respondents to adhere to norms of medical education in India, largely driven by their own past experiences. Some stakeholders had been trained in the Medical Council of India postgraduate system, and therefore wanted to uphold their perception of the normative process of generating specialists.

*“…that’s linked to fact that you know this chhaap (stamp) of a degree is very much in the DNA….because the degree is, you know, a thing of respect, it’s a strong social prestige factor”* [Former member of regulatory body].

International stakeholders also strongly supported this idea due to their own experiences. Establishing MD programs recognized by Medical Council of India is akin to initiating residencies in high-income countries; given the centrality of these programs to the development of the field in those countries, some respondents noted that international stakeholders were highly encouraging of this approach, and promoted it actively during conferences and other platforms.

*“…our goal was to encourage them to make it become an identified specialty to start specific residency programs in emergency medicine and to help pass on any lessons we have learnt over that 35 years of developing it in the United States”* [High-income country emergency physician].

Beginning in the late 1990s, SEMI leaders actively pursued this recognition, followed later by INDUS-EM leaders, Academy of Traumatology leaders and other actors working in an independent capacity. The role of these groups was all the more critical to the development of the field, due to a perceived lack of emphasis on specialty development within the Council.

*“…they (Medical Council of India) also will not do the groundwork to start a course. Someone else has to do that. But somebody needs to do the labor right? I mean the MCI wouldn’t go and initiate that leg work, spade work that you need…to take a call”* [Private sector stakeholder].

Stakeholders engaged the Council primarily through in-person meetings, letter writing, and invitations to Council leadership for emergency medicine conferences. Association leadership often led outreach efforts, with limited engagement from the broader membership of these associations. In terms of external framing of ideas around emergency medicine, stakeholders also largely had similar arguments, in terms of both the disease burden (increasing burden of non-communicable diseases and injuries) and service delivery challenges (poor quality of emergency care in hospitals). Advocacy efforts were uncoordinated, and each group pursued an independent course of action. Despite these myriad efforts, stakeholders were given limited indication about the views and actions within Medical Council of India on the request.

*“The advocacy was continuing, letters were going continuously, but MCI is a difficult body to work with, very difficult body to work with”* [Private sector stakeholder].

It was during this period of inaction from the Council in the 2000s that the solutions pursued by the emergency medicine network began to diverge. Stakeholders within SEMI and AAEMI felt that other options for postgraduate training were required to fill the gap in emergency physicians in India. These groups furthered their advocacy for specialty recognition with another regulatory body, the National Board of Examinations, which would allow for postgraduate training to formally begin in private hospitals. More importantly, the unregulated residency-style training programs that were initially set up between Indian hospitals and institutions in high-income countries began to flourish. These shifts towards regulated and unregulated private sector training exacerbated tensions between the professional groups. INDUS-EM stakeholders were of the belief that the NBE program should not outpace the growth of MD programs, while SEMI stakeholders felt that NBE programs were promising, given the active role of the private sector in the development of emergency medicine. From 2011 onwards, SEMI worked closely with the National Board to develop the program by actively participating in a transnational advisory council for the specialty. Some respondents, from INDUS-EM and to a lesser extent SEMI were also highly critical of the logistical and financial arrangements underlying the unregulated residency-style programs, noting that these programs were against the national interest and in the view of some, without sound legal basis. Other respondents believed that given perceived uncertainties within the Medical Council of India system, providing alternative training options was a key intervention in meeting the workforce shortages for emergency physicians.

*“Sometimes need outweighs regulation….the need is incredible. The need will not be served by simply the MEM, DNB and MCI programs alone. And so… I don’t know how that will fit into this equation. If your need is tremendous, because you are already starting behind, how do you make up that difference? As much as you say all the programs need to be India based, and I do believe that at some point that is important, you also have a large deficit that you need to make up. So that may also play a factor in the sustainability of some of these programs, in addition to the quality”* [High-income country emergency physician].

Finally, many respondents responded positively to the idea of emergency medicine as a concept emerging from high-income settings, while others were more skeptical of its applicability to the Indian context. More important however was the fact that some respondents alluded to an underlying lack of adaptation of emergency medicine to the Indian context. For example, one public sector respondent noted the need for a ‘purely Indianized’ form of emergency care, and others suggested that existing models of emergency medicine were perhaps not sufficiently adapted to India (for example, to rural healthcare).

*“So they used to come in every year, be there for the conference, talk about what they do in the US and then they would go back. I am not in favor of that, you see, because what happens is that you are trying to transplant a system and impose it in an alien environment, and it doesn’t really take root and it dies very quickly”* [Indian emergency physician].

### Political Context

Respondents converged around the idea of Medical Council of India recognition, but soon recognized the challenges that they would face in securing the recognition. By the early 2000s, the Council had evolved in increasingly political ways, and its leaders became embroiled in several corruption scandals.^63^ Advocacy for recognition by emergency medicine stakeholders therefore overlapped with a turbulent time at the Council. For example, in 2001-2002, the postgraduate committee did not meet for ten months due to a public interest lawsuit.^64^ Furthermore, several respondents noted that the 1990s and 2000s saw a shrinking of communication channels with Council administrators and an overall lack of prioritization of the institution to the issues of specialty development and recognition. Some respondents described becoming increasingly frustrated with the course of events, while a few others expected a drawn-out policy process.

The nature of bureaucracy within Medical Council of India also complicated matters, given the perceived lack of practical demarcations in responsibilities between executive leaders and the postgraduate committee during that time period, with respondents’ views mixed as to which group held final decision-making authority. Respondents also had to engage with the Council’s notoriously slow administrative system. Some respondents described the process of letter writing as a disheartening endeavor given the lack of response. A few respondents described in-person meetings as passive, often consisting of noncommittal respondents from Council administrators. Ultimately, few signals of the Council’s intentions were revealed until 2007-2008, when leaders told the Academy of Traumatology that they were interested in starting the program, and when a SEMI stakeholder was informed about the Council’s intentions.^65^ The lack of explicit guidelines or parameters for recognizing new medical specialties (by both the government and Medical Council of India) meant that decision-making was personalized. Current and former regulators noted that some of the reasons behind the approval were perceptions of increased private sector momentum, the pervasiveness of emergency medicine in high-income countries, demand for emergency medicine training programs within medical colleges, and perceptions regarding the national disease burden shifting towards increased non-communicable diseases and trauma-related injuries.

The policy window in this instance appeared to be less of a window caused by external factors, but rather, one where the convergence of multiple forces of advocacy applied enough pressure to facilitate the decision by Council leaders. Interestingly, action taken outside of SEMI and INDUS-EM appears to have helped facilitate a final decision. First, the National Human Rights Commission wrote to Medical Council of India requesting the recognition of emergency medicine as a medical specialty, eliciting a rare response on the subject in Council meeting minutes.^66^ Second, the Academy of Traumatology utilized its networks to access Council leadership to request recognition, so that planned postgraduate training programs in emergency medicine in Gujarat could get underway.

*“I would say window of opportunity, in one sense…that the time was right in so many frames, and MCI was primed, there were a lot of stakeholders, there was a demand for Medical Council to sort of start emergency medicine*...” [Private hospital stakeholder].

### Outcomes

Emergency medicine was formally notified by Medical Council of India on July 21, 2009 with the policy appearing in the Gazette of India.^67^ However, recognition proved to be the first of several steps in developing training programs in medical colleges, the details of which will be addressed in a forthcoming paper. Briefly, remaining issues included developing a standardized curriculum, finalizing eligibility criteria for faculty, and finalizing infrastructure requirements for starting programs. However, this next phase in the development of emergency medicine overlapped with a period of intense reform within Medical Council of India, following the arrest of its President in 2010.^68^ From 2010 to 2013, three separate Boards of Governors were installed to oversee the Council’s functioning; during this time, its leaders and administrators began to develop operational policies to guide training in the specialty. The National Board followed four years later by recognizing emergency medicine in 2013. That same year, representatives aligned with the Council’s previous leadership were installed into power.

The lack of cohesiveness within the emergency medicine network impacted the development of training policy. Starting from the 1990s, respondents from professional associations said that they repeatedly submitted curricula, faculty development plans, and other operational guidelines to Medical Council of India. However, stakeholders were not coordinated in their efforts, resulting in multiple versions of curricula and other training policies being shared. Ultimately, during the first Board of Governors phase in 2011, the leadership selected a group of four INDUS-EM members to design a curriculum (which was not released as of 2016). However, the first medical colleges to begin EM training were in Gujarat, and aligned with the Academy of Traumatology. Therefore, medical college programs appeared to develop somewhat independently, despite efforts of INDUS-EM to coalesce these programs (Observation data).

By the end of 2015, the impact of emergency medicine on the Indian landscape was coming into sharper relief. Emergency Departments began to emerge in urban and peri-urban centers around the country, offering more organized, and reportedly better quality, emergency care for patients, and serving as a key source of employment for new emergency physicians. Medical colleges around the country began offering emergency medicine training programs, and several other short-course training programs for general practitioners were being offered.^69,70^ In collaboration with an independent expert committee, the Ministry of Health and Family Welfare began designing a short-course training program in emergency care for frontline health workers in primary and secondary health facilities. Policy initiatives were also underway, such as certification standards for emergency departments in hospitals accredited by the National Accreditation Board for Hospitals and Healthcare Providers.^71^ However, network fragmentation in the efforts to improve hospital-based emergency care continued. For example, national-, state- and local-level partnerships to strengthen hospital-based emergency care continued to grow, independent of the main professional groups.^72-74^ Further, various conflicts were also heightened within the EM network. For example, long simmering tensions between SEMI and some high-income country stakeholders boiled over, resulting in a noticeably reduced presence of high-income country stakeholders in their 2015 national conference when compared to conferences in the 2000s (Observation data). Several medical college training programs faced regulatory hurdles with Medical Council of India, including the halting of formal recognition for several initiated programs in 2014-2015. The fate of unregulated programs also became increasingly contentious, with the Council ultimately clamping down in 2017 on certain programs.^75^ The competition between SEMI and INDUS-EM remained, limiting opportunities for coordination between public and private sector stakeholders. INDUS-EM also became a key partner to the National Board in 2015, diminishing SEMI’s role in developing those training programs (Observation data). SEMI and INDUS-EM also engaged in parallel policy efforts, including the introduction of national emergency care legislation (Observation data).^76,77^

## Discussion

Specialization in biomedicine has become globally ubiquitous, but the policy processes underlying specialization differ by country. We know little about how medical specialties emerge in LMICs, how they evolve in the context of health systems, and the resultant impact on equity.^25^ Our findings provide some of the first empirical research on medical specialization in India by using a political priority setting analysis to shed light on the emergence and recognition of new medical specialties. Here, we reflect on our key findings in the context of both the literature on political prioritization for health policies in LMICs, and on medical specialization, although the latter emanates largely from high-income settings.

The conceptual framework used for this study allowed for several insights into the policy process for medical specialization. In the category of actor power, we observed that the network seemed to comprise overlapping sub-networks – policy communities (professional association leadership), issue networks (members of professional associations or other interested individuals), and epistemic communities (transnational networks connecting Indian stakeholders with emergency physicians in high-income countries), resulting in an overall lack of cohesion, a reflection of the inefficiencies of India’s pluralistic system of organized medicine.^13^ In this case however, the lack of cohesion was not detrimental to achieving the stakeholders’ main policy objective – the recognition of emergency medicine. Consistent with other health policy analyses in LMICs,^34^ we find that the idea of emergency medicine specialization, rather than the policy community actors themselves, seems to have primarily motivated action on the part of regulator, perhaps due to the fact that the regulators were also medical professionals and therefore shared similar normative beliefs as those in the EM network. The power of this idea in influencing political priorities could have then been amplified by the diversity of voices in support, coming from the professional associations, the National Human Rights Commission and other concerned individuals (although important to mention, not noticeably from grassroots organizations). However, while the lack of policy community cohesion might not have had a negative impact on the main policy objective, it ultimately resulted in a fragmented and uncoordinated training landscape, and competing policy agendas between the professional associations. We suggest that policy community cohesion is therefore not only helpful for attaining primary objectives,^30,78^ but is also necessary to ensure a sustainable, coordinated long-term strategy for networks promoting new medical specialties.

Our analysis also suggests that actor power and national governance structures might interact in unpredictable, and potentially detrimental ways in the development of medical specialties in India. One of the key challenges in this case was the presence of an opaque governance structure in which stakeholders were operating. Rather than having an inchoate, regulatory system as seen early on in high-income settings,^8^ or the streamlined approaches that emerged later,^4,9^ the Indian regulatory system for medical specialization is highly bureaucratic but fragmented,^79^ creating multiple formal and informal pathways for specialist training and lacking a streamlined approach to specialty recognition. The relatively marginal role of the Indian government in postgraduate medical education has also limited the ability for the state to directly or indirectly intervene in prioritizing particular specialties, thereby placing further power in the hands of Medical Council of India, an institution with a seemingly problematic approach to developing specialization policy.^22^ The numerous challenges associated with the Council – bureaucratic delays, lack of communication channels, differing understandings of rules around postgraduate training policy – increase the chances for unintended, negative consequences for specialty development.^80^ Our case study clearly shows that groups within the emergency medicine network pursued myriad pathways for specialist training, immediately resulting in a lack of standardization and coordination, but also possibly resulting in negative downstream effects, such as poor availability and quality of care, on the Indian health system. Therefore, those policy communities advancing specialties that are operating in challenging governance contexts might require even more cohesion than those working in relatively transparent governance structures, in order to ensure coordinated, systematic approaches to advancing their objectives regarding the specialty.

Our findings also indicate the importance of the transnational epistemic community consisting of specialist Indian doctors and high-income country emergency physicians in advancing emergency medicine in India. International exchange has been highlighted as a major influence on the development of medical specialties in high-income countries, often through international meetings, exchanges and communication.^4^ However, our case indicates that high-income country stakeholders were more actively and directly involved in promoting specialties *within* India through these epistemic communities. In this, the role of the diaspora deserves particular attention. A key aspect of diasporic engagement is the idea of ‘social remittances,’^81^ or the exchange of ideas between their countries of residence and countries of origin. Medical specialization could be considered one such ‘remittance,’ given the role that diasporic engagement and transnationalism has played in influencing the growth of medical specialties in India.^26,82^ Such processes of diffusion appear to be stronger due to the transnational epistemic communities between doctors of particular specialties, such as surgery.^30^ In this case however, the involvement of the diaspora in professional associations, and in the development of emergency medicine more broadly in India, was both praised and criticized. These findings suggest that while this group has unique advantages in actively engaging in India (for example, cultural familiarity, language, and regional networks), their deep ties to India might on occasion cloud their willingness to introspect, and to fully appreciate the reasons behind resistance and backlash to their efforts.^83^ The power dynamics underlying the brokering of specialty development from high-income countries to LMICs, particularly in the context of technical expertise and financial power, warrants further attention.

Finally, the ideational centrality of postgraduate training in specialty development in India and other LMICs requires further interrogation. The literature on specialization from high-income settings indicates that obtaining formal recognition, and initiating and certifying training are primary goals for most specialties.^4,8^ However, a pluralistic, mixed health system requires more nuanced approaches that link efforts such as specialty development with other policies, such as training non-specialists across the system, in order to appropriately engage with issues of access and inequity.^84^ Therefore, we reason that relying primarily on postgraduate education might not sufficiently facilitate linkages with the broader health system, and therefore, may only benefit a minority of patients. Stakeholders seeking to make a broader impact through emergency medicine might consider proactively expanding interventions such as incorporating emergency medicine within the undergraduate medical curriculum, community-level programs, and training non-specialists in the health system.^19^

## Limitations

There were several limitations to this study. First, our study focuses on the single case of emergency medicine in India, and we encourage caution in applying these findings to other new medical specialties and other contexts, although we do believe that some commonalities might exist. Second, some respondents, particularly those involved with regulatory institutions, did not always recollect or reveal details of the prioritization of emergency medicine, and therefore, our data might reflect an incomplete understanding of the trajectory of the field. Third, we were unable to secure interviews from select government or regulatory stakeholders, and therefore, the findings might not fully incorporate potentially important perspectives, such as other viewpoints of leaders and administrators within regulatory institutions regarding specialty development. Fourth, while our sampling was comprehensive and reflected a diverse range of stakeholders, we were unable to interview all stakeholders involved in the development of emergency medicine in India since the early 1990s, and therefore, our findings might not capture certain perspectives. Finally, while we attempted to collect extensive documentary evidence, we were unable to secure certain documents, such as internal communications amongst stakeholders. We addressed these several of these limitations by comparing and triangulating data from the interviews, documents and observation, and validating our findings with key respondents.

## Conclusion

In this paper, we outlined the trajectory of a recent medical specialty in India over the last several decades, and examined the factors that influenced the development of policy for that field. We conclude that the development of medical specialization in India, particularly from a policy perspective, faces key challenges to coordination, standardization and growth, including a complex regulatory architecture, pluralistic organized medicine, the ideational centrality of postgraduate medical education, and poor linkages between specialization and the health system. Streamlining the process for both recognizing specialties, and standardizing training programs across medical institutions, could ensure improvements in availability, accessibility and quality of care. These concerns are not limited to India, and further empirical work is required to understand these issues in other LMICs. As medical specialization continues to shape and influence health systems globally, research exploring medical specialization can enhance our understanding of the linkages among policy, specialization, health systems, and equity.

## Acknowledgements

We thank the respondents for sharing their time and insight. We also recognize the support of the Centre of Social Medicine and Community Health at the Jawaharlal Nehru University (New Delhi, India) in conducting this study. Finally, we thank the anonymous peer reviewers for their detailed feedback.

## Ethical issues

The Institutional Review Board at Johns Hopkins Bloomberg School of Public Health provided ethical clearance for this study (IRB No. 00005860). An Ethics Committee of the Centre of Social Medicine and Community Health at Jawaharlal Nehru University, New Delhi, India reviewed study protocols and concurred with that decision.

## Competing interests

Authors declare that they have no competing interests.

## Authors’ contributions

VS and SB conceptualized and designed the study, with input from AAH. VS conducted all data collection. VS and SB analyzed the data. AAH contributed to the interpretation of the data. VS and SB drafted the manuscript, with critical revisions from AAH.

## Funding

Fieldwork for this study was supported by the American Institute of Indian Studies and the Department of International Health at the Johns Hopkins Bloomberg School of Public Health. VS is currently supported by the Agency for Healthcare Research and Quality under grant award T32 HS000087 (PI: Jane Holl, MD, MPH). Its contents are solely the responsibility of the authors and do not necessarily represent the official views of AHRQ.

## Authors’ affiliations

^1^Center for Health and the Social Sciences, University of Chicago, Chicago, IL, USA. ^2^Health Systems Program, Department of International Health and International Injury Research Unit, Johns Hopkins Bloomberg School of Public Health, Baltimore, MD, USA. ^3^Health Systems Program, Department of International Health, Johns Hopkins Bloomberg School of Public Health, Baltimore, MD, USA.

## Key messages

**Implications for policy makers**

- In India, inadequate linkages between specialized forms of healthcare found in tertiary settings and lower levels of the health system have long been identified. The findings of this study suggest that those weak linkages are exacerbated during the policy process to initiate medical specialties. The key stakeholders involved in medical specialization – regulators, the government, and professional associations – could take an early and active interest in discussing tangible efforts to ensure that possible benefits of specialization are woven into existing systems, particularly undergraduate medical training, primary care and community-based approaches.
- Stakeholders from high-income countries, particularly the Indian diaspora, strongly influence the development of medical specialization in India. However, few guidelines exist for their engagement, and the resulting ambiguity may have contributed to contested activities, such as the initiation of unregulated training programs. Stakeholders from high-income countries should ensure that they have a firm understanding of the legal, social, political, and economic landscape in which they are collaborating, and continuously reflect on their roles, in order to ensure that their partnerships remain equitable, productive and transparent.
- The complexities in the regulatory system for postgraduate medical education in India led to several different types of courses for emergency medicine in India, without adequate standardization or coordination. Streamlining the policy processes for both recognizing specialties, and standardizing training programs across medical institutions, could contribute to improvements in availability, accessibility and quality of care.

**Implications for the public**

The public are increasingly accessing specialized health services in low- and middle-income countries (LMICs), including India. However, we know little about how and why medical specialties emerge in India, and how those policy processes might potentially impact the Indian health system. In this study, we traced the trajectory of one recent medical specialty, emergency medicine, and found several challenges in the overall process to initiate new specialties in India, including fragmentation amongst specialist groups seeking to promote the specialty, a difficult regulatory environment in which to advance new specialties, a primary focus on postgraduate medical education, and weak linkages at the policy level between medical specialization and the broader health system. This case study indicates that policy-makers should consider streamlining the process of initiating medical specialties, so that the majority of the Indian public, particularly the poor, are better served by the potential benefits of medical specialization.
